# Improving the provision for gynaecological health care in Bangladesh using Essential Gynaecological Skills implementation package: a stakeholder-driven approach in public health facilities

**DOI:** 10.7189/jogh.15.04132

**Published:** 2025-05-05

**Authors:** Sabrina Jabeen, Mehedi Hasan, Santanu Acharya, Md Mahiur Rahman, Elizabeth Rafii-Tabar, Sarah Hall, Farhana Dewan, Azizul Alim, Sayeba Akhter, Shahin Rahman Chowdhury, Mustufa Mahmud, Md Jahangir Alam Prodhan, Salma Rouf, Sameena Chowdhury, SK Zinnat Ara Nasreen, Fawzia Hossain, Ferdousi Begum, Rubaiya Matin Chandrima, Md Akib Al-Zubayer, Golam Sarwar, Mahbuba Khan, Farida Akhter, Sayed Rubayet, Anisuddin Ahmed, Hassan Shehata, Shams El Arifeen, Ranee Thakar, Ahmed Ehsanur Rahman

**Affiliations:** 1 Maternal and Child Health Division, International Centre for Diarrhoeal Disease Research, Bangladesh (icddr,b), Dhaka, Bangladesh; 2 Royal College of Obstetricians and Gynaecologists, London, UK; 3 Obstetrical and Gynaecological Society of Bangladesh, Dhaka, Bangladesh; 4 Directorate General of Health Services, Ministry of Health and Family Welfare, Government of the People’s Republic of Bangladesh, Dhaka, Bangladesh; 5 Directorate General of Family Planning, Ministry of Health and Family Welfare, Government of the People’s Republic of Bangladesh, Dhaka, Bangladesh; 6 World Health Organization, Dhaka, Bangladesh; 7 Save the Children, Dhaka, Bangladesh; 8 Ipas Bangladesh, Dhaka, Bangladesh

## Abstract

**Background:**

Gynaecological health and its related service delivery have long been neglected in Bangladesh. In response to the high burden and improvements in the provision of gynaecological health care, the Essential Gynaecological Skills (EGS) implementation package was developed. It includes comprehensive in-service training for upskilling the non-specialised health care providers and introduces the first structured data recording system for gynaecology in the outdoor of public health facilities in Bangladesh. We outline how the stakeholder engagement process was integrated into the implementation research related to this pioneering initiative.

**Methods:**

Based on literature review, expert consultation and prior experience, we adopted the identification, sensitisation, involvement, and engagement (ISIE) framework of stakeholder engagement and process documentation. After identifying national and local level stakeholders via a power-interest mapping exercise, we sensitised them to the gaps in gynaecological health service delivery. High-power and high-interest stakeholders were involved and engaged in developing the EGS implementation package, which was then introduced in selected public health facilities and evaluated through implementation research.

**Results:**

Acknowledging the urgent need for gynaecological health care services, the identified and sensitised stakeholders supported the development of the EGS implementation package. This resulted in the development and implementation of the EGS implementation package under the Government of Bangladesh’s leadership, highlighting government ownership. These outcomes reflected the potential for scalability and sustainability of the package. However, stakeholder engagement remains a time and resource-intensive process that requires an innovative, research-backed approach with committed implementation.

**Conclusions:**

Our experience of using the ISIE framework showcased the potential of this framework in achieving sustainability and scalability at the national level. However, further initiatives from the government can ensure nationwide scale-up, setting an example for other lower and middle-income countries.

Gynaecological diseases contribute significantly to the global burden of disease, affecting millions of women worldwide and leading to considerable morbidity and mortality [1]. Whilst there has been significant progress in women’s health globally over the last two decades, much of the focus has been on maternal health, which has resulted in gynaecological health remaining under-prioritised despite an enormous burden of mortality and morbidity [1–3]. Globally, among women aged 15–49 years, years lived with disability (YLD) caused by benign gynaecological conditions (BGC) was 6.66% and due to maternal health conditions was 0.48% in 2001. The substantial difference in YLD remained nearly unchanged over the next two decades, reaching 6.28% for BGCs and 0.38% due to maternal health conditions in 2021 [1]. Among women aged 15–49 years in low- and middle-income countries (LMICs), YLD due to BGCs was 6.64% in 2001 and minutely reduced to 6.31% in 2021. This demonstrates a low prioritisation of BGCs, which has significantly worsened the burden of unnoticed yet preventable morbidities and contributed to a poor quality of life for women and girls, particularly in LMICs [1,4].

Similarly, in Bangladesh, YLD due to BGC’s was 5.08% in 2001, and by 2021, this had become 4.93% in Bangladesh. Over the last two decades, the Government of Bangladesh has significantly improved women’s health care. This includes sector programs, policies, strategies, guidelines, and standard operating procedures to enhance maternal health care services nationwide [5–9]. The result of these initiatives is reflected in a significant reduction of the maternal mortality ratio from 322 to 196 per 100 000 live births according to the national survey data, whereas YLD due to maternal conditions decreased from 0.81% to 0.31% two decades [1,10–14]. Despite sporadic data sources suggesting a high prevalence of BGCs in Bangladesh, there is no comprehensive national data source to prove the evidence nationally due to the absence of a structured data recording system for gynaecological diseases [15,16]. There have been multiple structured registers allocated to record information of obstetric patients in different service delivery points of public health facilities, whereas it is non-existent for gynaecological services. Even in the sector programmes of the Government of Bangladesh, the majority of the activities are maternal health-centric, and only a few vertical initiatives are there to address the gynaecological issues [5,6].

To address the gaps in the gynaecological services in the LMIC, the Royal College of Obstetricians and Gynaecologists has developed a comprehensive training package under their Gynaecological Health Matters programme [17]. The programme offers extensive training on essential gynaecological skills specifically to upskill the non-specialist health care providers to manage gynaecological health conditions in outdoor health care facilities [17].

As the Royal College of Obstetricians and Gynaecologists training modules were generic, country-context adaptation was done, and the Essential Gynaecological Skills (EGS) implementation package was developed with the training programme as one of its core components. Integrating the EGS implementation package into routine outdoor gynaecological services necessitated a comprehensive understanding of the unique perspectives, challenges, and opportunities in the Bangladeshi health care context. The Maternal Health Programme of Bangladesh led this initiative with funding from Global Affairs Canada and the Else Kröner-Fresenius-Stiftung Foundation. Technical support was provided by the Royal College of Obstetricians and Gynaecologists and the Obstetrical and Gynaecological Society of Bangladesh. icddr,b managed the project's operations and conducted implementation research in four Dinajpur and Kushtia districts public facilities. The Government of Bangladesh's ownership was secured through extensive stakeholder engagement to ensure scalability and sustainability.

Stakeholder engagement played a crucial role in adapting the Royal College of Obstetricians and Gynaecologists EGS training modules and developing an implementation package by fostering collaboration, balancing power dynamics, and ensuring that research is relevant and aligned with practical needs [18–21]. Ultimately, the whole process led to the effective implementation of evidence-based practices and improved health outcomes. This paper aims to demonstrate the rationale, strategy and process of a context-driven stakeholder engagement process and the impact of the policy and programme at the national level.

## METHODS

Stakeholder engagement is a complex process and is often tailored to specific priorities. Various frameworks to approach stakeholder engagement in health research have been reported in the literature [22]. These methods include the identification, sensitisation, involvement, engagement (ISIE) framework, the community-based participatory research, the participatory action research, the responsibility, accountability, consulted, and informed matrix, the Delphi method, the World Health Organization/World Economic Forum (WHO/WEF) stakeholder model, and the reconceptualised life-cycle theory among others [23–30].

**Figure 1:**
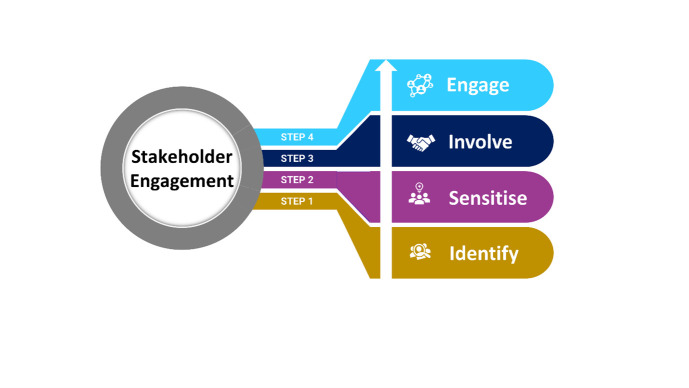
Conceptual framework adapted to guide the stakeholder engagement process to integrate EGS implementation package in routine gynaecological health care services in Bangladesh.

In our study, based on previous experience, consultation with policymakers and subject matter experts and gathering practical insights, we opted for the four sequential steps of the ISIE stakeholder engagement model as it is more flexible compared to other models (**Figure 1**) [31,32].

### Adaptation of a conceptual framework of stakeholder engagement for EGS implementation package

To introduce the EGS implementation package in Bangladesh, involving stakeholders, ongoing dialogue was required to seek their perspectives, knowledge, and values to establish common ground, align with project objectives, and enable transparent decision-making [22]. By adopting the ISIE method as a conceptual framework, efficient stakeholder identification, sensitisation, involvement, and engagement were ensured for successful implementation and sustainability of the training initiative.

### Step one: identifying stakeholders for sensitisation, involvement, and engagement

To comprehensively understand the stakeholders involved in implementing obstetrical and gynaecological health care services in Bangladesh, we reviewed pertinent documents (Table S1 in the **Online Supplementary Document**). We identified the wings of the Government of Bangladesh working with women’s health, including reproductive and adolescent health [33,34]. We also identified the development partners and professional societies working with the Government to manage and implement maternal health care services in public health facilities [33].

Additionally, we strategically chose and interviewed 10 key informants to pinpoint stakeholders at both district and national levels who were working with a vision to improve maternal or women’s health in the country. Consequently, we identified 10 national-level and 17 district-level stakeholder organisations, encompassing government health programs, professional bodies, United Nations (UN) agencies, local, national, and international organisations, as well as non-government organisations (NGO) dedicated to gynaecological health in Bangladesh.

**Figure 2:**
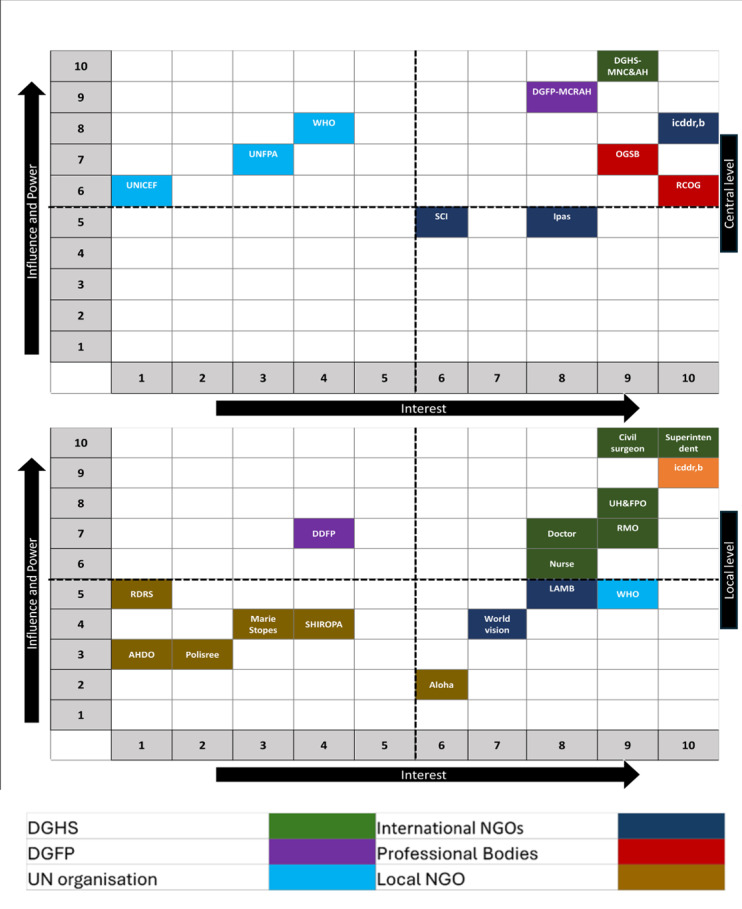
Power-interest mapping of national and district-level stakeholder organisations related to the EGS implementation package development and implementation in Bangladesh.

To maximise the use of limited resources, priority was given to a specific group of stakeholder organisations that aligned closely with the project's objectives. To facilitate this process, we convened a consultative workshop with the concerned authorities within the government of Bangladesh. During this workshop, participants, including the line director, programme managers and deputy programme managers of the maternal health programme of the Directorate General of Health Services (DGHS) utilised a 10-point Likert scale to develop the power-interest matrix (**Figure 2**) for prioritising stakeholder organisations based on their influence/power and interest in gynaecological health care service (Table S2 in the **Online Supplementary Document**) [35,36]. Stakeholders were categorised into groups based on their average interest and power scores. Organisations with an interest score of six or higher were considered to have high interest, while those with a power score of six or higher were classified as having high power. Out of the 10 national-level stakeholders identified, we categorised five as high-power-high interest groups, three as high-power-low interest groups, and two as low-power-high interest groups.

Among the 17 district-level stakeholders identified, seven were placed in the high-power-high-interest group as they were striving to enhance maternal health services in the Kushtia and Dinajpur districts.

We applied a similar power-interest matrix to health care providers. From both matrices, it was determined that the maternal health programme at DGHS and Directorate General of Family Planning (DGFP), along with icddr,b, and health professionals from these organisations, exhibited the highest level of commitment and cooperation. We opted to notify (outlined in step two) all stakeholders with significant power or interest while directing our efforts towards selecting stakeholders with both high power and high interest during the involvement stage (outlined in step three) and the engagement phase (outlined in step four).

### Step two: sensitising stakeholders for awareness building

The primary goal was to persuade the identified stakeholders to acknowledge the existing gaps in gynaecological health care and the EGS implementation package as a suitable and affordable solution. A central-level workshop was arranged to sensitise stakeholders at the national level, aiming to foster understanding and collaboration among key actors in the field. In the workshop, the maternal health programme of DGHS and DGFP, professional bodies like Obstetrical and Gynaecological Society of Bangladesh and Royal College of Obstetricians and Gynaecologists, development partners including WHO, United Nations Population Fund (UNFPA), United Nations Children’s Fund (UNICEF), Ipas Bangladesh, Save the Children Bangladesh, and others participated. The programme manager of the maternal health programme, DGHS, chaired the sessions to ensure country ownership and government leadership. The icddr,b research team led discussions on the burden of gynaecological diseases in Bangladesh, barriers and enablers in service provision, and the importance of EGS training for non-specialist doctors and nurses, highlighting its alignment with the Bangladesh Essential Service Package (Table S3 in the **Online Supplementary Document**) [37].

We held two sensitisation workshops in the Kushtia and Dinajpur districts. Participants included district health managers (civil surgeons), hospital superintendents, assistant directors, and sub-district health managers (Upazila health and family planning officers). Gynaecological service providers, including consultants, non-specialist physicians, and nurses, also attended. After focusing on the gaps in the gynaecological service delivery, the discussion was followed by a project description and its implementation plans. Finally, we organised two additional sensitisation workshops at the district levels led by the implementation facility managers, with representatives from 31 local-level NGOs of Kushtia and Dinajpur districts, who were involved in gynaecological services.

### Step three: involving stakeholders in joint planning and sharing responsibilities

Through a series of joint planning sessions, stakeholders delved into the outlining of the prerequisites and strategised the feasibility assessment of the EGS implementation package in Bangladesh.

In the central level planning and consultative workshop held in December of 2022, we discussed the gaps in gynaecological service delivery and data recording systems. The main priorities were the development of the EGS implementation package, providing training to upskill the non-specialised health care provider on gynaecological service delivery through training programmes and the development of a structured data recording system in the outdoor service delivery. To carry forward these activities, the maternal health programme of the DGHS agreed to lead the project, and a technical working group was formed under their leadership with the representatives from DGHS, DGFP, Obstetrical and Gynaecological Society of Bangladesh, WHO, UNICEF, UNFPA, Ipas Bangladesh, Save the Children and icddr,b.

In the consultative workshop, we selected Kushtia and Dinajpur district hospitals and Daulatpur and Fulbari Upazila health complexes (sub-district level facilities) as implementing facilities. To design, develop, and implement the EGS package in Bangladesh, we defined the roles and responsibilities of collaborating partners. icddr,b was assigned to coordinate the overall process and act as the primary liaison among stakeholders at international, central, and local levels.

As decided in the consultative workshop, we organised four planning meetings at the facility level to discuss the training programmes, encompassing schedule, trainers, duration, and methodology, to guarantee the successful training on the EGS implementation package to nurses and non-specialist doctors. Moreover, with the meetings, we aimed to identify suitable locations within the outdoor health care facilities for service delivery, optimising space and resources to provide efficient gynaecological care. We also debated the allocation of trained personnel, such as physicians and nurses, within health care facilities, as well as the formulation of a pairing of trained doctor and nurse strategy to maximise cooperation and ensure comprehensive coverage of gynaecological services. All the facility managers agreed upon this ‘pairing model’ of service delivery.

### Step four: engaging stakeholders in design, development, and implementation

The terms of reference of the technical working group included the adoption of EGS training modules and the development of the data recording system. Through a series of meetings, a technical working group designed and developed the EGS implementation package and provided consultative support for field implementation as needed.

National-level involvement in the implementation research was crucial for designing and overseeing the training and deployment of the EGS implementation package. This involvement included the selection and training of master trainers and expert trainers, as well as the development and adaptation of training modules and materials. Furthermore, national-level organisations were key actors in providing guidance and support throughout the implementation process.

The implementation outcome of the EGS implementation package was agreed to be measured with both quantitative and qualitative data using the WHO implementation research variables [38]. It was decided that the EGS training would be evaluated using the four-level evaluation framework adapted from the WHO training evaluation guideline [39].

The technical working group concluded that a comprehensive documentation system would be required for this package. Thus, documents were developed focusing on the capacity development of the non-specialised health care providers for gynaecology outdoor, job aids for facilitation of day-to-day activities, data recording systems for health management information system integration, and frameworks for monitoring and supervision. The entire document development process involved the following events. Country adaptation of the EGS training modules – the original training modules developed by the Royal College of Obstetricians and Gynaecologists were adapted in alignment with the prevailing standards and guidelines endorsed by the government of Bangladesh. Selected master trainers from the Obstetrical and Gynaecological Society of Bangladesh took the lead for the clinical part, and the rest of the members from the technical working group were thoroughly updated according to the implementation perspective. The EGS modules cover eleven gynaecological topics (**Table 1**).

**Table 1:** Topics covered by the EGS training and facilitator modules

Topics
Basic reproductive sciences
Contraception
Sexually transmitted infections and HIV
Emergency gynaecology
Abnormal uterine bleeding
Cervical cancer
Infertility
Early pregnancy loss
Urogynaecology
Clinical audit
Violence against women

Development of job aids – relevant visual information and infographics were noted during the country adaptation of the modules, which could serve as valuable resources for aiding non-specialised health care providers in informed decision-making during gynaecological service delivery. It was decided that these job aids would be distributed and placed in gynaecology outdoors to facilitate accessibility and utilisation by health care providers.

Data recording system development – initially, the icddr,b research team, comprising physicians and anthropologists, visited selected implementing facilities to assess patient flow to identify the various layers or routes of services that patients with gynaecological complaints received. Subsequently, the existing unofficial and non-structured data recording system for gynaecological patients was examined, and health care providers were engaged to understand their opinions and expectations regarding the structured gynaecology outpatient register. Leveraging this information and using the existing government health care registers (*e.g.* Emergency Obstetric and Newborn Care register, antenatal/postnatal care register) as reference points, necessary variables were identified for the gynaecological register based on the consensus of the technical working group members. To ensure user-friendliness and straightforwardness of the gynaecological register, diagnoses were streamlined, and diseases were categorised following expert consultation. Quality assurance testing was conducted to maintain reliability and usability throughout the development process.

The development of additional materials for the data recording system, including a structured referral form for gynaecology outdoors and a monthly reporting form to collate data on gynaecological cases, were convened simultaneously. Moreover, the monitoring process by the maternal health programme, DGHS, was strengthened by the inclusion of indicators on gynaecological conditions in the existing monitoring checklist.

**Figure 3:**
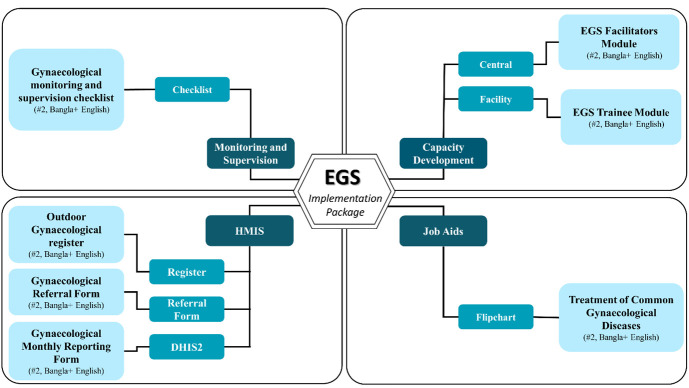
Documents developed for the EGS implementation package for improving the gynaecological services in the outdoors of public health facilities.

Translation of the EGS implementation package – after finalising the country adaptation, the EGS modules (both training and facilitator modules) underwent translation into Bengali by anthropologists. The translated modules were meticulously reviewed and double-checked by physicians from the research team to ensure accuracy in medical terminology and jargon. Similarly, the documents for the data recording system were also translated into Bengali. Once the EGS modules and the entire data recording system were finalised, they were extensively evaluated and endorsed by the senior leads of the Maternal Health Programme and other stakeholder organisations for implementation in Government health facilities. The package consists of a total of 14 documents (**Figure 3**; Table S4 in the **Online Supplementary Document**).

Upon approval, the EGS implementation package was introduced for the capacity building of health care providers, followed by the introduction of the data recording system in the implementing facilities to document data regarding gynaecological cases effectively. The training for the EGS implementation package was organised in two stages following a casket pattern.

Initially, we conducted a training of the trainers in Dhaka, led by 14 master trainers who were senior obstetricians and gynaecologists selected from the Royal College of Obstetricians and Gynaecologists and the Obstetrical and Gynaecological Society of Bangladesh. We formed a pool of a total of 26 expert trainers who were selected by senior consultants of the Obstetrical and Gynaecological Society of Bangladesh, consisting of mid-level gynaecologists who were members of the Obstetrical and Gynaecological Society of Bangladesh. Subsequently, expert trainers transmitted the training to non-specialist physicians and nurses at the facility level. Healthcare providers were trained in two batches spanning three days each, with 76 individuals trained across district hospitals and Upazila health complexes.

Additionally, we conducted a refresher training at implementation facilities after three months post-main training, with 73 participants. The training materials, including EGS training modules and related documents, were consistently utilised across all training stages. Throughout, icddr,b facilitated training sessions, ensuring effective dissemination of essential gynaecological health care skills and knowledge among health care providers.

A total of 12 champions from the health care providers were identified based on their exceptional performance during EGS training. Subsequently, gynaecological working groups were formed in each facility, comprising an obstetrics and gynaecology consultant, EGS-trained doctors and nurses tasked with advancing EGS package implementation. The consultant, serving as an expert trainer, oversaw regular monitoring of registers, referral forms, and monthly reporting while maintaining communication with facility managers for effective service delivery. The icddr,b field team provided support to these groups and communicated their service delivery needs to the central research team, facilitating bi-weekly case review sessions led by the obstetrics and gynaecology consultant and bi-monthly performance appraisal sessions led by the facility managers.

Local-level involvement in the implementation research focused on establishing gynaecological working groups within health care facilities at the district and Upazila/sub-district levels. They played a crucial role in monitoring the completion of registers, referral forms, and monthly reporting forms. This local-level engagement ensured that the implementation of the EGS package was tailored to the specific needs and contexts of each health care facility.

### Resources required for stakeholder engagement

The total resources required for stakeholder engagement-related activities of the EGS implementation package are presented in Figure S1 in the **Online Supplementary Document**.

### Desk review

We allocated a total of 312 person-days to the meticulous review of 118 documents spanning approximately 8239 pages. Physicians with fundamental gynaecological training who leveraged existing knowledge to enrich the assessment process undertook the process.

### Resource person interview

We conducted 10 interviews with prominent policymakers and influential figures in the field of women’s health within the country to pinpoint and prioritise relevant stakeholders. The entire process, including preparation, interviews, transcription, and analysis, demanded a cumulative effort of 63 person-days to accomplish.

### EGS implementation package documents development

Throughout the process of the EGS implementation package development, we dedicated an extensive effort of a total of 1199 person-days to meticulously planning, designing, developing, reviewing, and addressing various aspects of the package. The package itself comprised a total of 14 documents, each carefully crafted to facilitate the effective implementation of the EGS initiative.

### Workshops

The process of sensitising (step two), involving (step three), and engaging (step four) stakeholders required a total investment of 1779 person-days through 74 workshops. This collaborative effort involved both central and local level icddr,b staff, with 539 person-days contributed by the central team and 1269 person-days by the local team.

### Travel and transport

A combined total of 626 person-days were dedicated to extensive travel and transportation. Central staff required 271 days of travel, while local staff needed 359 days for visits to the four implementing facilities and within Dhaka.

### Timeline

The time required for the stakeholder engagement activities and important milestones achieved during that time are described in Figure S2 in the **Online Supplementary Document**.

### Step one: identify

We commenced the identification process in May 2022 and concluded it in April 2023. Nationally, this step extended from May 2022 to December 2022, while at the district level, it persisted from August 2022 to April 2023.

### Step two: sensitise

The sensitisation phase commenced in August 2022 and concluded in May 2023. It occurred in August 2022 at the national level and spanned from March to May 2023 at the district level.

### Step three: involve

This process spanned from December 2022 to November 2023. At the national level, the involvement process occurred from December 2022 to October 2023, whereas at the district level, it continued from May to November 2023.

### Step four: engage

The engagement process commenced in March 2023 and reached its culmination in November 2023. It occurred from March 2023 to April 2024 at the national level. Meanwhile, at the district level, engagement commenced in May 2023 and terminated in November 2023.

## RESULTS

### Impact of the stakeholder engagement process

#### Ownership at the national policy level

Acknowledging the importance and urgency of addressing the gaps in gynaecological service delivery, the Government of Bangladesh and all the stakeholders agreed to develop and implement the EGS implementation package. As a reflection of the ownership and interest, the Maternal Health Programme of DGHS immediately agreed to lead the project.

#### Technical working group formation at the national level

Under the leadership of the maternal health programme, DGHS, we formed a technical working group with the members of the high-power and high-interest group of stakeholders. With their active engagement, the EGS implementation package was developed and endorsed nationally, and a total of 102 health care providers were trained on it.

#### Initiatives towards scalability and sustainability

The remarkable response and participation of the government and central-level and facility-level stakeholders have proven the potential of the EGS implementation package in ensuring the delivery of outpatient gynaecological services in public health facilities. As a result, the government of Bangladesh has proposed including the EGS implementation package to scale-up in Bangladesh's next five-year sector programme.

#### Lessons learned

The complex interplay of a multitude of factors that determined the outcome of this stakeholder engagement procedure, ultimately bringing success, is presented in the strength, weakness, opportunity and threat analysis (Figure S3 in the **Online Supplementary Document**) approach [40,41].

First strength was a groundbreaking notion. The pioneering innovation of addressing the long-overlooked need for comprehensive gynaecological training for non-specialised health care providers and a data recording system for gynaecological services in the outdoor public health facilities gained the attention and interest of the stakeholders. Further, strengths included expertise of the facilitating organisation. icddr,b has a long-standing reputation for implementation research with the country's health system along with vast experience in working with multiple national and international partners. These recognitions and experiences helped in liaising with the stakeholders. Moreover, alignment with the national policy is another strength. The EGS implementation package addresses common gynaecological conditions treatable in outpatient settings in Bangladesh and aligns with most Bangladesh Essential Service Package components, which facilitates the Government and other stakeholders' engagement (Table S3 in the **Online Supplementary Document**). The research-backed approach was another strength. icddr,b has effectively applied the ISIE model of stakeholder engagement in implementation research previously and succeeded in fostering trust and acceptance. icddr,b’s facilitation, communication, organisational skills, and strong reputation have been key contributors to EGS’s success.

One weakness was a resource-demand, *i.e.* we required a considerable amount of person-time involvement of the research team members and the stakeholders was the literature review, meetings and workshops. In addition to financial, logistics and extensive human resource support, the research team required considerable person-days of travelling. Further, starting from the desk to the implementation in the facilities, it took around two years of stakeholder engagement activities requiring uninterrupted commitment from the facilitating organisation. Moreover, the training module adaptation process required the focused attention of the research team and technical working group members for the extensive review of all related national program documents. The development of a data recording system in consonance with the national data flow structure involved elaborate consultation among the stakeholders for five months. Effective team engagement was required to avoid the stakeholders losing interest and withdrawing from the stakeholder team. Further, bringing together various stakeholders led to personal or organisational interests colluding. Organising training sessions involving stakeholders at both central and local levels resulted in tension among them on occasions where the facilitating organisation had to act as a mediator. Therefore, effective communication and predicting sensitive situations were imperative for stakeholder coordination.

Opportunities were related to increasing awareness regarding sexual and reproductive health. There is a growing interest in policy-level decisions and programs prioritising sexual and reproductive health rights. The Government of Bangladesh has given importance to the activities related to sexual and reproductive health rights in its future five-year sector program, thereby reinforcing the position of the EGS implementation package.

Threats were related to political instability. Political instability or shifts in government priorities could disrupt program continuity and funding, jeopardising long-term sustainability. From October 2023 to January 2024, supervision visits between Dhaka and implementing facilities were hindered by strikes and blockades related to political unrest during the national election. Threats were also associated with leadership transitions. The maternal health programme, DGHS, experienced leadership transitions, including changes in the position of line director twice and a mid-implementation transition in the role of deputy programme manager and alongside turnover in pivotal positions at the district level, such as the superintendent of Kushtia general hospital, required significant efforts by the project team to acclimate new appointees to project objectives. Further, priorities in health service delivery focused primarily on maternal health. Additionally, cultural reluctance to seek gynaecological health care posed an initial challenge.

## DISCUSSION

### Importance of stakeholder engagement

In implementation research and policy change initiatives, stakeholder engagement is essential for achieving long-term objectives. Stakeholder engagement fosters ownership and sustainability by involving local and central stakeholders in the decision-making process, which enhances their commitment and support for the initiative [20,42–44]. The EGS implementation package development is an ideal example in this regard. As the government and stakeholders recognised the urgent need to address gaps in gynaecological service delivery, they agreed upon the necessity and relevance of the EGS package as a long-term solution. The commitment of high-level stakeholders resulted in the formation of a maternal health programme-led technical working group, the national endorsement of the EGS package, and the systematic training of 102 health care providers. Similar to our endeavour, the success of the community clinic model in Bangladesh demonstrates that ownership, sustained support and sustainability for health care initiatives can be fostered through the extensive involvement of local-level stakeholders [43]. The Community Access to Child Health (CATCH) study in Kenya and the National Institute for Health and Care Research Global Health Research Unit on Respiratory Health (RESPIRE) project in South Asia both reported that enhancing scalability and translating research into policy and practice resulted from tailoring interventions to the local context through stakeholder engagement [20,44]. Similar to our experience, extensive SE aids in aligning interventions with local priorities, leading to more effective resource allocation, as seen in maternal and child diet-improving initiatives in sub-Saharan Africa and Asia [45].

### Consequences of inadequate stakeholder engagement

Ill-defined objectives result from inadequate stakeholder engagement, which in turn can produce negative program outcomes [46]. Negligence in stakeholder engagement often results in a gap in the understanding of local cultural and socioeconomic contexts. This misalignment also hampers efforts to influence policies, as a clear understanding is essential for aligning initiatives with existing policies. Without the alignment of the EGS implementation package with the existing Bangladesh Essential Service Package and the national data flow structure, gaining the attention of the stakeholders would have been very unlikely. Findings from a study conducted by Mariam et al. mirror our experience as interventions lacking local stakeholder insight may not be adequate for diverse and dynamic contexts [42]. This was evident when in 2010, a Kenyan research group was attacked by the local community as an outburst of extreme distrust resulting from contrasting cultural approaches [47].

Leadership transitions within the focal roles of government greatly disrupt and threaten the continuity of the project as we have seen during this implementation research. Consistent stakeholder engagement enabled us to make timely measures to acclimate the new stakeholders to the objectives and importance of this initiative. Similarly, Sobrinho et al. reported that the dynamics of policy implementation were influenced by the changes in the position of power further highlighting the importance of stakeholder engagement [48].

However, competing health interests can divert the focus of the stakeholders from gynaecological health, particularly in Bangladesh, where maternal and child health is prioritised. Recent studies in Bangladesh and other countries found that the focus of the stakeholders shifted greatly towards the COVID-19 pandemic, limiting attention to other health care services [49–51]. Therefore, without extensive stakeholder engagement, gynaecological health would receive insufficient attention from Bangladesh’s resource-limited health care system.

### The role of organisational capabilities

The success of implementation research can partially be attributed to the capabilities of the implementing organisations. Factors such as experience in implementation, relationships with national and international stakeholders, and the use of effective stakeholder engagement models are significant for fruitful implementation [52,53]. icddr,b, with its long-standing collaboration with the Government of Bangladesh, international organisations, and development partners, has extensive experience working in maternal health and navigating the public health system [54]. By addressing the need for gynaecological training for non-specialised health care providers and introducing a data recording system, icddr,b gained stakeholder interest in effectively improving the long-overlooked gynaecological health system in Bangladesh. Its proven expertise in stakeholder engagement along with strong facilitation and communication skills, fostered trust and collaboration [31,32,55]. This foundation of experience contributed to the successful implementation of the EGS package, resulting in national endorsement and stakeholder commitment.

### Why did we choose ISIE

Although stakeholder engagement is an undeniable contributor to the success of any implementation research, the effectiveness can vary significantly depending on the adopted method [18,22]. Approaches like community-based participatory research, participatory action research, and reconceptualised life-cycle theory build trust and relevance among stakeholders but are resource-intensive, lengthy, and may overlook key stakeholders due to their bottom-up approach [23–26]. Tools like the responsibility, accountability, consulted, and informed matrix clarify roles but lack flexibility, while the Delphi method and WHO/WEF model support structured decision-making but are time-consuming and ill-suited for rapid public health needs [21,27–30].

The ISIE framework, in contrast, is particularly suited for the EGS package due to its iterative nature, which allows for real-time adjustments based on feedback and evolving circumstances [31,32]. ISIE’s structured stages ensure that stakeholder engagement translates into targeted outcomes, addressing immediate project goals and effectively involving multiple levels of stakeholders. Its modular and scalable nature also facilitates the expansion and adaptation of the EGS package across diverse regions while supporting sustainable engagement [21,31,32].

The paper has several strengths, including stakeholder involvement in the manuscript writing, which enhances credibility. It effectively highlights the importance of SE in implementation research, supported by evidence from successful health initiatives. The focus on local context and cultural factors emphasises the need for tailored interventions. However, as the implemented stakeholder engagement model specifically focuses on health care initiatives, it may not fully address the applicability of the model for interventions in different contexts.

## CONCLUSIONS

Our experience from the stakeholder engagement by using the four-stage ISIE model has showcased its potential to bring attention to neglected gynaecological health. Starting with a comprehensive training program, this engagement process led to the establishment of an implementation package for gynaecology. This initiative not only sheds light on gynaecological health but also underscores the potential of the EGS implementation, resulting in the integration of the package into the next five-year sector programme of the Government of Bangladesh. Although the ISIE model is time-consuming and resource-intensive, this model can be used in different countries with proper conceptualisation coupled with adequate time, resources, commitment and collaboration among stakeholders. However, further initiatives by the Government, including the stakeholders, can ensure the smooth scale-up of the EGS implementation package in Bangladesh. The experience of integrating the ISIE model synergistically with the EGS implementation package can be an example for other low and middle-income countries in advancing their provision for gynaecological health services.

## Additional material


Online Supplementary Document
