# Supplementary material for: Improving the provision for gynaecological health care in Bangladesh using Essential Gynaecological Skills implementation package: a stakeholder-driven approach in public health facilities
Source: J Glob Health. 2025 May 5;15:04132. doi: 10.7189/jogh.15.04132 (PMC12050113; doi:10.7189/jogh.15.04132)

Supplement to: Jabeen S, Hasan M, Acharya S, Rahman MM, Rafii-Tabar E, Hall S, Dewan F, Alim A, Akhter F, Akhter S, Rahman Chowdhury S, Mahmud M, Alam Prodhan MJ, Rouf S, Chowdhury S, Nasreen SKZA, Hossain F, Begum F, Chandrima RM, Al-Zubayer MA, Sarwar G, Khan M, Akhter F, Rubayet S, Ahmed A, Shehata H, El Arifeen S, Thakar R, Rahman AE. Improving the provision of gynaecological healthcare in Bangladesh using the Essential Gynaecological Skills implementation package: a stakeholder-driven approach in public health facilities. J Glob Health. 2025;15:04132.

Table S1: Documents reviewed for developing EGS implementation package

SL	Name of the Document	Page Count
1	An investigation into the relationship between training evaluation and the transfer of training	10
2	Adaptation of Kirkpatrick's Four-Level Model of Training Criteria to Evaluate Training Programmes for Head Teachers	25
3	Training Evaluation for Nurses and Midwives Using the Kirkpatrick Model: A Qualitative Descriptive Study	14
4	Evaluation Of Medical Teacher’s Training Program Through Kirkpatrick’s Model	5
5	Research methodology workshops evaluation using the Kirkpatrick’s model: Translating theory into practice	7
6	Harnessing power of simulation training effectiveness with Kirkpatrick model in emergency surgical airway procedures	7
7	The effectiveness of in-service training “pharmacopeia home health” based on Kirkpatrick’s model: A quasi-experimental study	5
8	Employing Kirkpatrick’s Evaluation Framework to Determine the Effectiveness of Health Information Management Courses and Programs	5
9	Using Kirkpatrick’s model to measure the effect of a new teaching and learning methods workshop for health care staff	5
10	Evaluation of infection prevention and control training workshops using kirkpatrick’s model	5
11	Medical Education & Family Welfare (ME&FW) Division, Ministry of Health and Family Welfare, Government of the People's Republic of Bangladesh.	1
12	Allsop, D. B., Chelladurai, J. M., Kimball, E. R., Marks, L. D., & Hendricks, J. J. (2022). Qualitative Methods with Nvivo Software: A Practical Guide for Analyzing Qualitative Data. Psych, 4(2), 142-159. https://www.mdpi.com/2624-8611/4/2/13	17
13	Assessment of Healthcare Providers in Bangladesh 2021. (2021).	92
14	Balen, A. H. (2017). Polycystic ovary syndrome (PCOS). The Obstetrician & Gynaecologist, 19(2), 119-129.	10
15	Bangladesh Essential Service Package (ESP). In: Ministry of Health and Family Welfare	53
16	Bangladesh National Strategy for Maternal Health 2019-2030. (2019). In: Directorate General of Health Services (DGHS),	90
17	Bangor, A., Kortum, P. T., & Miller, J. T. (2008). An empirical evaluation of the system usability scale. Intl. Journal of Human–Computer Interaction, 24(6), 574-594.	22

18	Bray, F., Ferlay, J., Soerjomataram, I., Siegel, R. L., Torre, L. A., & Jemal, A. (2018). Global cancer statistics 2018: GLOBOCAN estimates of incidence and mortality worldwide for 36 cancers in 185 countries. CA: a cancer journal for clinicians, 68(6), 394-424.	30
19	Brooke, J. (1996). SUS-A quick and dirty usability scale. Usability evaluation in industry, 189(194), 4-7.	7
20	Coco, A. S. (1999). Primary dysmenorrhea. American family physician, 60(2), 489.	1
21	Comprehensive cervical cancer control: a guide to essential practice. (2006). World Health Organization.	408
22	da Fonseca, M. H., Kovaleski, F., Picinin, C. T., Pedroso, B., & Rubbo, P. (2021). E-health practices and technologies: a systematic review from 2014 to 2019. Healthcare,	32
23	Davis, F. D. (1989). Perceived Usefulness, Perceived Ease of Use, and User Acceptance of Information Technology MIS Quarterly, 13(3), 319-340. https://doi.org/10.2307/249008	16
24	El-Habibi, M. F., Megdad, M. M., Al-Qadi, M. H., AlQatrawi, M. J., Sababa, R. Z., & Abu-Naser, S. S. (2022). A Proposed Expert System for Obstetrics & Gynecology Diseases Diagnosis.	17
25	Ferlay, J., Shin, H. R., Bray, F., Forman, D., Mathers, C., & Parkin, D. M. (2010). Estimates of worldwide burden of cancer in 2008: GLOBOCAN 2008. International journal of cancer, 127(12), 2893-2917.	25
26	Gillenson, M. L., & Sherrell, D. L. (2002). Enticing online consumers: an extended technology acceptance perspective. Information & management, 39(8), 705-719.	15
27	Hammond, R. (2002). Gynaecological causes of abdominal pain. Surgery (Oxford), 20(7), 173-176.	3
28	Human Resources for Health (HRH) Project in Bangladesh Final Report July 2012 - December 2016.	46
29	Iyoke, C. A., & Ugwu, G. O. (2013). Burden of gynaecological cancers in developing countries. World Journal of Obstetrics and Gynecology, 2(1), 1-7.	7
30	Khan, A. W. (2010). Continuing Professional Development (CPD); What should we do? . Bangladesh Journal of Medical Education, 1(1), 37-44.	8
31	MIS, D. (2019). Health Bulletin 2019.	263
32	National Institute of Population Research and Training (NIPORT), & ICF. (2020). Bangladesh Demographic and Health Survey 2017-18.	511
33	Ng, A. W. Y., & Lo, H. W. C. Measuring the Usability of Safety Signs : A Use of System Usability Scale (SUS).	6
34	Obstetrical and Gynecological Society of Bangladesh (OGSB). Current OGSB Projects. https://www.ogsb.org/web_admin/page/Activity/ogsb-projects	1
35	Population & Housing Census 2022 Preliminary Report. (2022).	68
36	Spagnoletti, B. R. M., Bennett, L. R., Keenan, C., Shetty, S. S., Manderson, L., McPake, B., & Wilopo, S. A. (2022). What factors shape quality of life for women affected by gynaecological cancer in South, South East and East Asian countries? A critical review. Reproductive Health, 19(1), 70.	20
37	Vaessen, M. (1984). Childlessness and infecundity.	46

38	Weiers, R. (2011). Introduction to business statistics, South-WesternCengage Learning", New York, USA.	892
39	Weström, L. (1991). Pelvic inflammatory disease. JAMA, 266(18), 2612-2612.	1
40	Wijeratne, D., & Fiander, A. (2018). Gynaecological disease in the developing world: a silent pandemic. The Obstetrician & Gynaecologist, 20(4), 237-244. https://doi.org/https://doi.org/10.1111/tog.12515	8
41	World Health, O. (2010). Evaluating training in WHO. In. Geneva: World Health Organization.	68
42	Sonalkar S, Gaffield ME. Introducing the World Health Organization postpartum family planning compendium. International Journal of Gynecology & Obstetrics. 2017 Jan;136(1):2-5.	5
43	Family Planning:A global handbook for providers. 3rd edition, 2018. WHO, USAID https://www.fphandbook.org/sites/default/files/global-handbook-2018-full-web.pdf	442
44	Family Planning Manual 2017, DGFP,MOHFW, supported by USAID & EngenderHealth, Bangladesh	413
45	CCSDP Unit of DGFP, MOHFW. Family Planning Handbook for Medical Students and Physicians [Internet]. 2020 [cited 2023 Dec 7]. Available from: https://dgnm.portal.gov.bd/sites/default/files/files/dgnm.portal.gov.bd/page/82b7e673_6743_4d1d_88af_cb21167eb5d3/2022-01-05-08-01-0a56746ced98ee08ac32281773a7793c.pdf	129
46	National Guidelines For Management Of Sexually Transmitted Infections (Third Edition)	192
47	How health providers can support women who have experienced violence [Internet]. Who.int. [cited 2023 Dec 7]. Available from: https://www.who.int/westernpacific/news-room/multimedia/overview/item/how-health-providers-can-support-women-who-have-experienced-violence	1
48	Violence against women [Internet]. Who.int. [cited 2023 Dec 7]. Available from: https://www.who.int/news-room/fact-sheets/detail/violence-against-women	1
49	Engaging with stakeholders for community-based health research in India: Lessons learnt, challenges and opportunities	5
50	Engaging stakeholders in implementation research: lessons from the Future Health Systems Research Programme experience	3
51	Achieving impact: exploring the challenge of stakeholder engagement	11
52	Lessons Learned From the Stakeholder Engagement in Research: Application of Spatial Analytical Tools in One Health Problems	9
53	Big data and predictive analytics in healthcare in Bangladesh: regulatory challenges	12
54	Health system in Bangladesh: Challenges and opportunities	9
55	An Analysis of the Problems for Health Data Integration in Bangladesh	4
56	Evaluating Hospital-Based Surveillance for Outbreak Detection in Bangladesh: Analysis of Healthcare Utilization Data	18

57	Perceptions and experiences with district health information system software to collect and utilize health data in Bangladesh: a qualitative exploratory study	4
58	Health Data Integration with Secured Record Linkage	6
59	Production and use of estimates for monitoring progress in the health sector: the case of Bangladesh	11
60	Towards development of health Data Warehouse: Bangladesh perspective	6
61	RCOG Essential Gynaecological Skill: Facilitator Module	186
62	RCOG Essential Gynaecological Skill: Trainee Module	200
63	WHO Recommendation for The Prevention and Treatment of Postpartum Haemorrhage (PPH)	48
64	Eclampsia and PPH Action Plan in Bangladesh	42
65	Guideline on Intrapartum care (IPC) and Postnatal Care (PNC)	66
66	Guidelines of Paper Based Emonc Register	4
67	Bangor A, Kortum PT, Miller JTJIJoHCI. An empirical evaluation of the system usability scale. 2008;24(6):574-94.	20
68	Joo HJIJoAER. A study on understanding of UI and UX, and understanding of design according to user interface change. 2017;12(20):9931-5.	5
69	Ng AW, Lo H, Chan A, editors. Measuring the Usability of Safety Signs: A use of system usability scale (SUS). proceedings of the International MultiConference of Engineers and Computer Scientists; 2011: Citeseer.	7
70	Brooke J. System Usability Scale (SUS): A quick and dirty usability scale. 1996.	8
71	Bangor A, Kortum PT, Miller JT. An empirical evaluation of the system usability scale. Int J Hum Comput Interact. 2008;24(6):574-94.	22
72	Davis FD. A technology acceptance model for empirically testing new end-user information systems: Theory and results: Massachusetts Institute of Technology; 1985.	290
73	Davis FDJMq. Perceived usefulness, perceived ease of use, and user acceptance of information technology. 1989:319-40.	24
74	Gillenson ML, Sherrell DLJI, management. Enticing online consumers: an extended technology acceptance perspective. 2002;39(8):705-19.	15
75	Research NIoP, Training - NIPORT, Health Mo, Family Welfare, ICF. Bangladesh Demographic and Health Survey 2017-18. Dhaka, Bangladesh: NIPORT/ICF; 2020.	92
76	Mehra D, Sarkar A, Sreenath P, Behera J, Mehra SJBph. Effectiveness of a community based intervention to delay early marriage, early pregnancy and improve school retention among adolescents in India. 2018;18(1):1-13.	13
77	Joshi S. Female household-headship in rural Bangladesh: incidence, determinants and impact on children's schooling. 2004.	50
78	UNICEF. The state of the world's children 2007: women and children: the double dividend of gender equality: Unicef; 2006.	160
79	The System Usability Scale: Past, Present, and Future	24
80	A review of technology acceptance and adoption models and theories	8
81	Investigating acceptance of telemedicine services through an extended technology acceptance model (TAM)	10

82	Technology acceptance model: a literature review from 1986 to 2013	12
83	Technology Acceptance Model in M-learning context: A systematic review	16
84	Acceptability of healthcare interventions: an overview of reviews and development of a theoretical framework	13
85	Measuring AT Usability with the Modified System Usability Scale (SUS)	23
86	A Systematic Review of the Technology Acceptance Model in Health Informatics	8
87	Investigating acceptance of telemedicine services through an extended technology acceptance model (TAM)	10
88	Nurses' perceptions, acceptance, and use of a novel in-room pediatric ICU technology: testing an expanded technology acceptance model	15
89	The Usage Behavior and Intention Stability of Nurses: An Empirical Study of a Nursing Information System	33
90	Acceptability of healthcare interventions: an overview of reviews and development of a theoretical framework	13
91	Maternal Health Standard Operating Procedure (SOP) Volume 1	226
92	Maternal Health Standard Operating Procedure (SOP) Volume 2	384
93	Bangladesh National Strategy for Maternal Health (English)	90
94	Bangladesh National Strategy for Maternal Health (Bangla)	90
95	Amulya J. What is reflective practice. Center for Reflective Community Practice, Massachusetts Institute of Technology, Cambridge, MA[Online] Available at: http://www itslifejimbutnotasweknowit org uk/files/whatisreflectivepractic e pdf [Accessed 15 April 2009]. 2004.	5
96	Directorate General of Health Services MoHaFW. 4th Health, Population and Nutrition Sector Program (4th HPNSP), January 2017 - June 2022. 2017.	284
97	Maternal Health Programme DGoHS, Ministry of Health and Family Welfare. National Maternal Health Programme Implementation Toolkit. 2022. Available: https://resourcecentre.savethechildren.net/pdf/maternal-health-program-implementation-toolkit-2022.pdf/. Accessed.	120
98	Te Braak P, van Tienoven TP, Minnen J, Glorieux I. Data quality and recall bias in time-diary research: The effects of prolonged recall periods in self-administered online time-use surveys. Sociological Methodology. 2023;53:115-38.	35
99	DHIS2 Central database [Internet]. [cited 13 October 2023]. Available from: https://dghs.portal.gov.bd/site/page/492255ac-64e8-4a48-ac34-173ebe6a4290.	1
100	Implementation research for developing Civil Registration and Vital Statistics (CRVS) Systems: lessons from Indonesia	12
101	Rahman AE, Jabeen S, Fernandes G, Banik G, Islam J, Ameen S, et al. Introducing pulse oximetry in routine IMCI services in Bangladesh: a context-driven approach to influence policy and programme through stakeholder engagement. Journal of Global Health. 2022;12.	14
102	Rahman AE, Ameen S, Hossain AT, Perkins J, Jabeen S, Majid T, et al. Introducing pulse oximetry for outpatient management of childhood pneumonia: An implementation research adopting a district implementation model in selected rural facilities in Bangladesh. EClinicalMedicine. 2022;50.	17

103	Nuswantari A, Wu Y-T, Surjono HD, editors. System Usability Scale Measurement on Synhcronous Online Argumentation Learning System. International Conference on Online and Blended Learning 2019 (ICOBL 2019); 2020: Atlantis Press.	5
104	Frøkjær E, Hertzum M, Hornbæk K, editors. Measuring usability: are effectiveness, efficiency, and satisfaction really correlated? Proceedings of the SIGCHI conference on Human Factors in Computing Systems; 2000.	8
105	Bogren M, Erlandsson K, Byrskog U. What prevents midwifery quality care in Bangladesh? A focus group enquiry with midwifery students. BMC health services research. 2018;18:1-9.	9
106	Franklin M. The nurse-midwifery challenge: Bridging the technology gap. Journal of Nurse-Midwifery. 1994;39:110-1.	2
107	Mohamamad A, Yunus AM. Technology Acceptance in Healthcare Service: A Case of Electronic Medical Records (ERM). International Journal of Academic Research in Business and Social Sciences. 2017;7:863-77.	15
108	Kalayou MH, Endehabtu BF, Tilahun B. The applicability of the modified technology acceptance model (TAM) on the sustainable adoption of eHealth systems in resource-limited settings. Journal of multidisciplinary healthcare. 2020:1827-37.	11
109	Tubaishat A. Perceived usefulness and perceived ease of use of electronic health records among nurses: Application of Technology Acceptance Model. Informatics for Health and Social Care. 2018;43:379-89.	10
110	Paulsen A, Overgaard S, Lauritsen JM. Quality of data entry using single entry, double entry and automated forms processing–an example based on a study of patient-reported outcomes. PloS one. 2012;7:e35087.	6
111	Larrabee JH, Boldreghini S, Elder-Sorrells K, Turner Z, Wender R, Hart J, et al. Evaluation of documentation before and after implementation of a nursing information system in an acute care hospital. Computers in nursing. 2001;19:56-65; quiz 6.	9
112	National Institute of Population Research and Training MEaFWD, Ministry of Health and Family Welfare, Dhaka, Bangladesh, The DHS Program, ICF, Rockville, Maryland, USA. Bangladesh Demographic and Health Survey, Key indicators report. 2022.	84
113	Jabeen S, Siddique AB, Hossain AT, Khan S, Haider MM, Tahsina T, et al. Haemorrhage-related maternal mortality in Bangladesh: Levels, trends, time of death, and care-seeking practices based on nationally representative population-based surveys. Journal of Global Health. 2023;13.	11
114	National Institute of Population Research Training (NIPORT) ICfDDR, Bangladesh, MEASURE Evaluation. Bangladesh Maternal Mortality and Health Care Survey 2016 Final Reprot. 2017. Available: http://rdm.icddrb.org/wp-content/uploads/2020/04/BMMS-2016-Final-Report_10-Feb-2020.pdf. Accessed: 25 October, 2021.	361
115	National Institute of Population Research and Training (NIPORT) MoHaFWMF. Bangladesh Health Facility Survey 2017 Final Report. 2020. Available: https://dhsprogram.com/pubs/pdf/SPA28/SPA28.pdf. Accessed.	318

116	World Health Organization. Active management of the third stage of labour-New WHO recommendations help to focus implementation. 2014. Available: https://www.who.int/reproductivehealth/publications/maternal_perinatal_health/new-recommendations-amtsl/en/. Accessed: 03 November, 2021.	2
117	The National Institute of Population Research and Training (NIPORT) MEaFW, Division MoHaFW, The DHS Program, ICF. Bangladesh Demographic and Health Survey 2017-18. 2017-18. Available: https://dhsprogram.com/pubs/pdf/PR104/PR104.pdf. Accessed: 26 October, 2021.	511
118	Systematic Process Framework for Conducting Implementation Science Research in Food Fortification Programs	9
**Total**		**8239**

Table S2: Power-Interest matrix scores of the stakeholders

SL	Acronym	Stakeholder	Level	Power	Interest
1	DGHS-MNC&AH	Directorate General of Health Services- Maternal Newborn Child & Adolescent Health	National	10	9
2	DGFP-MCRAH	Directorate General of Family Planning- Maternal Child Reproductive & Adolescent Health	National	9	8
3	icddr,b	International Centre for Diarrhoeal Disease Research	National	8	10
4	OGSB	Obstetrical and Gynaecological Society of Bangladesh	National	7	9
5	RCOG	Royal College of Gynaecology	National	6	10
6	WHO	World Health Organization	National	8	4
7	UNFPA	United Nations Population Fund	National	7	3
8	UNICEF	United Nations International Children's Emergency Fund	National	6	1
9	Ipas	Ipas Bangladesh	National	5	8
10	SCI	Save the Children International	National	5	6
11	Superintendent	Superintendent of District Hospital	District	10	10
12	Civil Surgeon	Civil Surgeon of the District	District	10	9
13	icddr,b	International Centre for Diarrhoeal Disease Research	District	9	10
14	UH&FPO	Upazilla Health and Family Planning Officer	District	8	9
15	RMO	Residential Medical Officer	District	7	9
16	Doctor	Medical Officer	District	7	8
17	Nurse	Nurse	District	6	8
18	DGFP	Directorate General of Family Planning	District	7	4
19	WHO	World Health Organization	District	5	9
20	LAMB	Lutheran Aid to Medicine in Bangladesh	District	5	8
21	World Vision	World Vision	District	4	7
22	Aloha	ALOHA Bangladesh- Local Non-Government Organisation	District	2	6
23	RDRS	Rangpur Dinajpur Rural Service-Local Non-Government Organisation	District	5	1

24	Shiropa	Shiropa Development Society- Local Non-Government Organisation	District	4	4
25	Marie Stopes	Local Non-Government Organisation	District	4	3
26	Pollisree	Local Non-Government Organisation	District	3	2
27	AHDO	Action for Human Development Organization- Local Non-Government Organisation	District	3	1

Table S3: Mapping of EGS topics with Essential Service Package components

Si no	EGS topics	ESP components	ESP sub-components
1.	Basic reproductive sciences and clinical skills	-	-
2.	Contraception	• Family Planning	○ Pre-conception<br>○ Post-partum<br>○ Post-abortion<br>○ Post-MR
3.	Sexually transmitted infection and HIV	• Communicable diseases	○ HIV/AIDS
		• Maternal, newborn, child and adolescent health care	○ Adolescent health: Adolescent sexual and reproductive health
4.	Emergency gynaecology and the management of the acutely ill women	-	-
5.	Abnormal uterine bleeding (AUB)	-	-
6.	Cervical cancer	• Non-Communicable Diseases	○ Cervical cancer<br>- Screening for cervical cancer (VIA)<br>- Colposcopic Examination (excision and biopsy) and cryotherapy
7.	Early pregnancy loss	• Family Planning	○ Post-abortion<br>○ Post-MR
8.	Subfertility	-	-
9.	Urogynaecology	• Maternal, newborn, child and	○ Postnatal care:

		adolescent health care	- Early management of obstetric fistula<br>- Management of genital prolapse
10	Improving standards; an introduction to clinical audit	-	-
11	Gender-based violence (GBV)	• Non-Communicable Diseases	○ Sexual and gender-based violence

Table S4: List of documents in the EGS implementation package

SL	Name of the Documents	No of pages
1	EGS trainee module - English	300
2	EGS trainee module - Bangla	312
3	EGS Facilitator module - English	284
4	EGS Facilitator module - Bangla	274
5	Gynaecological Service Register for Hospital Outdoor Use - English	3
6	Gynaecological Service Register for Hospital Outdoor Use - Bangla	3
7	Gynaecological Service Monthly Reporting Form - English	1
8	Gynaecological Service Monthly Reporting Form - Bangla	1
9	Gynaecological Services Referral Form - English	1
10	Gynaecological Services Referral Form - Bangla	1
11	Gynaecological Supervision and monitoring Checklist - English	3
12	Gynaecological Supervision and monitoring Checklist - Bangla	3
13	Job Aid - English	10
14	Job Aid - Bangla	10
**Total**		**1206**

Figure S1: Resources required for the stakeholder engagement activities to integrate the EGS Implementation Package for improving the gynaecological services in the outdoors of the public facilities

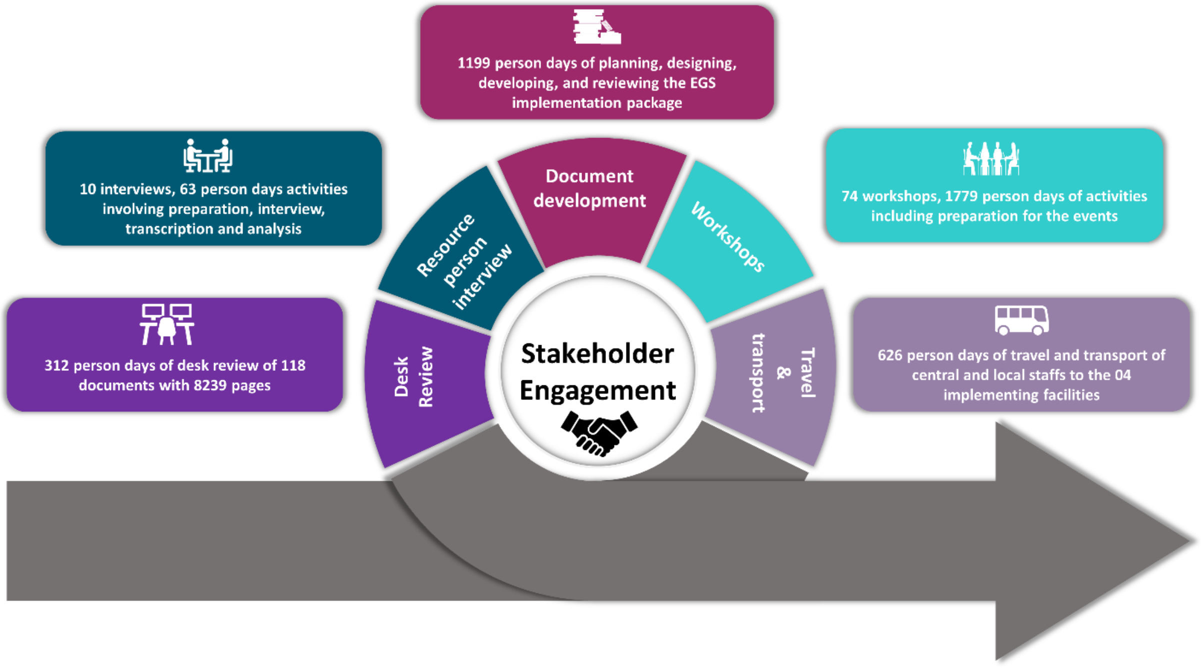

Figure S2: Timeline and milestones of the stakeholder engagement activities

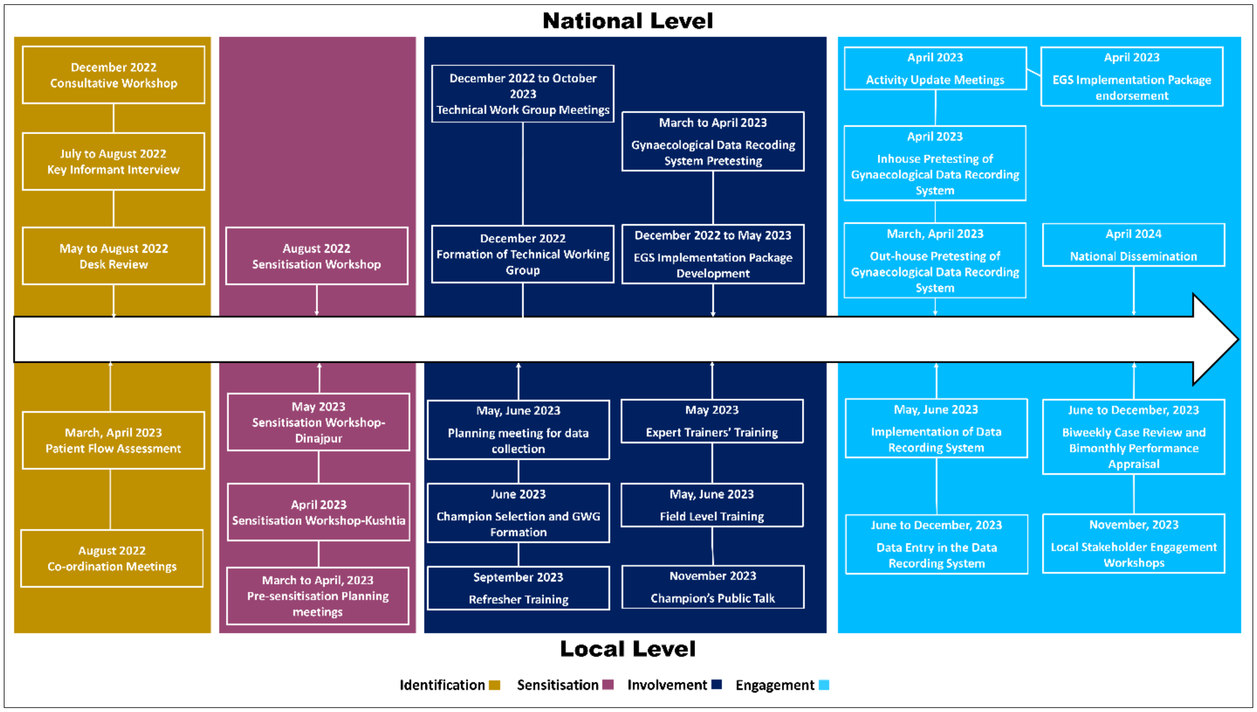

Figure S3: Strength, weakness, opportunity and threat analysis of the SE process for the EGS implementation package

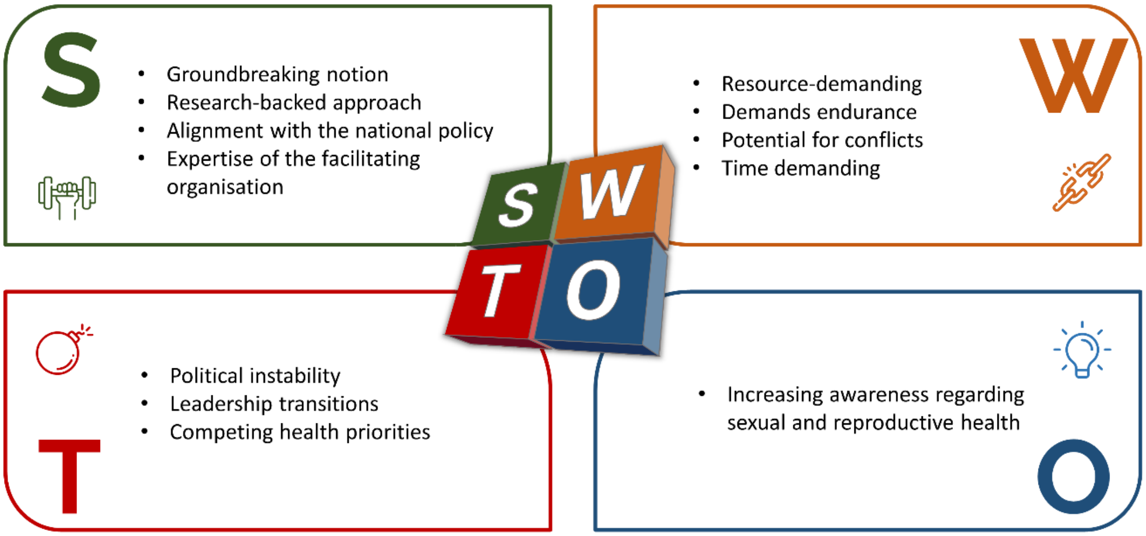

Supplement: Online Supplementary Document [file jogh-15-04132-s001.pdf]
